# An Unusual Cause of Cerebellitis in an Immunosuppressed Elderly Man: Disseminated Scedosporium boydii Following Gardening-Related Injury

**DOI:** 10.7759/cureus.87529

**Published:** 2025-07-08

**Authors:** Abdullah Abdullah, Yomon Jasim, Mohamed Alagili

**Affiliations:** 1 General Medicine, Barnet Hospital, Royal Free London NHS Foundation Trust, London, GBR; 2 Geriatrics, University Hospitals Dorset NHS Foundation Trust, Bournemouth, GBR

**Keywords:** cellulitis, cerebellitis, corticosteroid use, disseminated fungal infection, fungal cns infections, immunosuppression, opportunistic fungal infections, scedosporium boydii

## Abstract

*Scedosporium boydii* is an uncommon but increasingly recognized cause of opportunistic infections, particularly in immunocompromised individuals. We describe the case of a 92-year-old man on long-term corticosteroids who presented with progressive gait instability, confusion, and posterior headache. Initial neuroimaging revealed parietal-occipital and cerebellar lesions, raising concerns for malignancy or infarction. Although corticosteroid therapy initially provided temporary symptom relief, the patient subsequently deteriorated. Skin changes consistent with cellulitis were developed in the upper limb, and rapid progression of neurological lesions on repeat imaging, findings more suggestive of an infectious rather than a malignant process. Cultures from the skin lesion grew *S. boydii*, prompting the initiation of voriconazole and flucytosine. Despite appropriate antifungal therapy, the patient’s condition worsened, culminating in multiorgan failure and death. Retrospective history revealed a fall in a muddy garden, suggesting direct cutaneous inoculation as the source of infection, an atypical presentation since most cases are associated with near-drowning incidents. This case highlights the diagnostic challenges posed by rare fungal central nervous system infections that mimic more common intracranial pathologies and underscores the importance of early tissue sampling and multidisciplinary collaboration in managing such complex cases.

## Introduction

*Scedosporium boydii* and *S. apiospermum* are environmental molds that are increasingly recognized as significant opportunistic pathogens in immunocompromised hosts [1]. These fungi are found in soil, stagnant water, and decaying organic matter, with infections typically occurring through traumatic inoculation, inhalation, or near-drowning incidents [1]. While localized cutaneous infections are the most common presentation, invasive infections can involve the central nervous system (CNS) and carry a high mortality rate [1,2].

This report describes a rare case of cerebellitis and CNS involvement by *S. boydii* following a gardening-related injury in an elderly immunosuppressed patient. This unusual presentation highlights the importance of considering fungal infections in patients with atypical neurological symptoms and a history of environmental exposure.

## Case presentation

A 92-year-old gentleman presented to the hospital with a three-day history of severe, predominantly posterior headache and slurred speech. His wife reported a five-week history of progressive gait instability, confusion, and increasing difficulty standing independently. Previously, he had been fully independent, driving until shortly before admission and using a walking stick for support.

His past medical history included type 2 diabetes mellitus managed with oral hypoglycemics, atrial fibrillation, prior transient ischemic attack, coronary artery bypass grafting, amputation of the left lateral toes, and giant cell arteritis. His regular medications included finasteride, esomeprazole, folic acid, calcium, simvastatin, aspirin, and prednisolone. 

Notably, he had an unwitnessed fall five weeks earlier while gardening, landing in a muddy bush. He sustained only minor scratches at the time and had no immediate functional limitations.

On initial examination, he was afebrile, alert, and hemodynamically stable (National Early Warning Score or NEWS: 0). Neurological examination revealed confusion, significant dysarthria with a likely element of dysphasia, and bilateral upper and lower limb ataxia, more marked on the right. Bilateral nystagmus was present but no facial weakness. Pupils were equal and reactive but small. No focal limb weakness was noted; power, reflexes, and sensation were otherwise normal, and plantars were downgoing. There were no skin rashes or stigmata of infective endocarditis, though bruising was noted. Fundoscopic examination was limited. The patient reported blindness in his right eye due to giant cell arteritis eight years before.

Initial blood tests showed mild anemia and mild renal impairment, and C-reactive protein (CRP) was <1. Table 1 shows blood results on admission.

**Table 1:** Blood test results on admission ALP, alkaline phosphatase; ALT, alanine transferase; CRP, C-reactive protein; GFR, glomerular filtration rate; WCC, white cell count

Parameter	Result	Normal range
Total WCC	16	4.0-10.0 * 10^9/L
Neutrophil count	11.3	2.0-7.0 * 10^9/L
Lymphocyte count	0.4	1.0-3.0 * 10^9/L
Hemoglobin	118	130-170 g/L
Platelet count	224	150-410 * 10^9/L
Serum urea	9.5	2.5-6.7 mmol/L
Serum creatinine	84	59-104 µmol/L
GFR (mL/min/1.73 m^2^)	69	-
Serum sodium	132	132-146 mmol/L
Serum potassium	4.5	3.5-5 mmol/L
Serum albumin	27	29-45 g/L
Serum ALT	19	0-35 U/L
Serum ALP	35	30-150 U/L
Serum bilirubin	6	0-17 µmol/L
CRP	<1	0-9 mg/L

**Figure 1:**
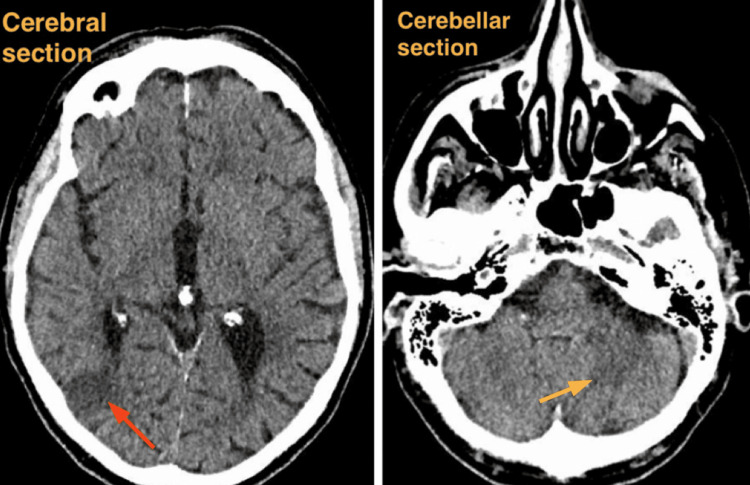
Non-contrast CT head on admission. Cerebral section on the left, and cerebellar section on the right On the left, cerebral section showing right temporal-occipital lesion. On the right, cerebellar section showing smaller lesions in the right cerebellar hemisphere and an ill-defined area of low density in the left cerebellar hemisphere. The red arrow is pointing to the site of cerebral lesion in the right temporal-occipital region. The yellow arrow is pointing to the site of the left cerebellar hemisphere lesion.

A non-contrast CT head on admission revealed a 2 cm intraparenchymal lesion in the right temporal-occipital region with surrounding vasogenic edema, effacing the right occipital horn and trigone. Additional smaller lesions were noted in the superior right cerebellar hemisphere, and an ill-defined low density was observed in the superior left cerebellar hemisphere. No acute hemorrhage or extra-axial collection was seen. Figure 1 shows the CT image on admission, showing both cerebral and cerebellar sections.

**Figure 2:**
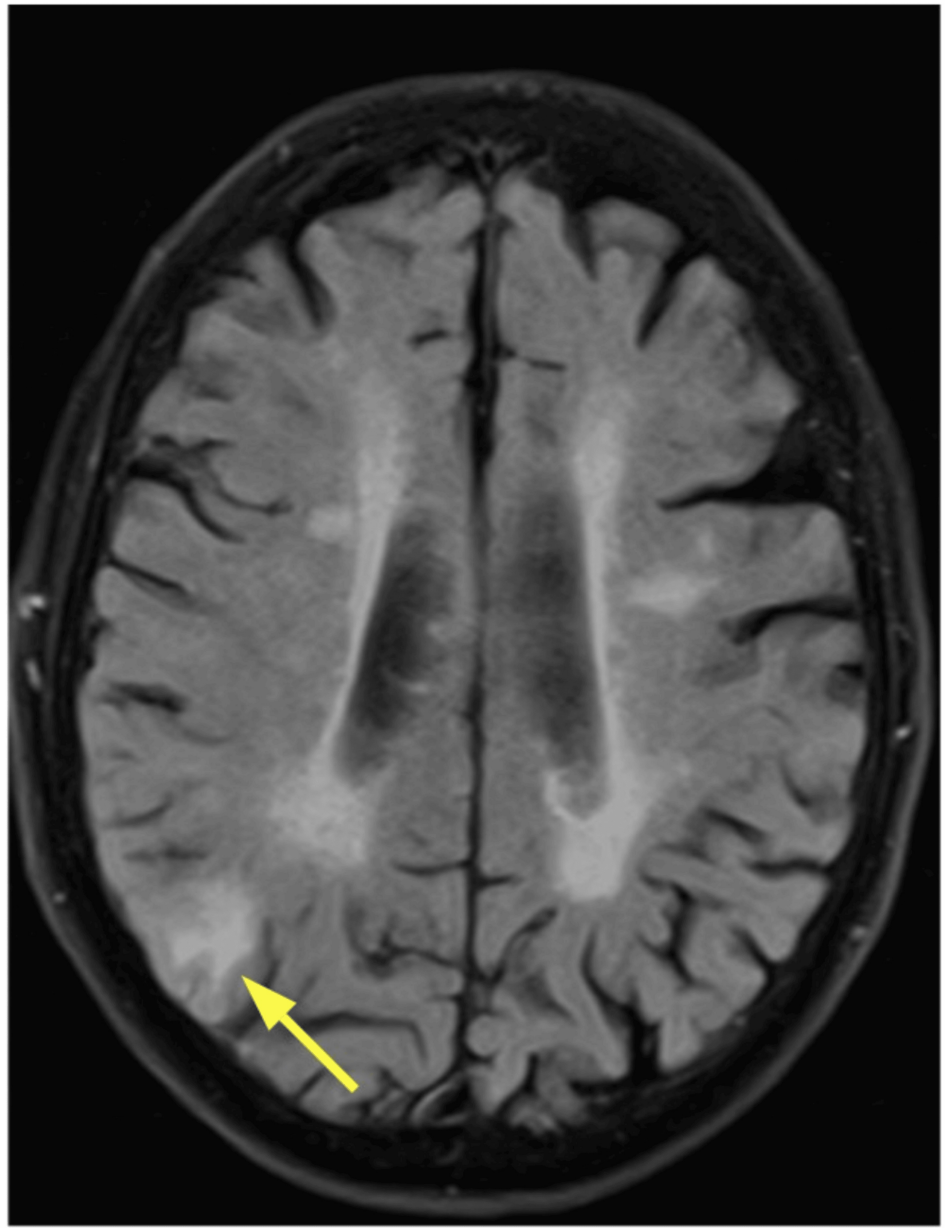
MRI brain: cerebral section Within the right parieto-occipital region is an approximately 2 cm area of cortical/subcortical enhancement. There is surrounding white matter vasogenic edema and local mass effect with effacement of adjacent sulci and right occipital horn. The yellow arrow is pointing to the lesion in the right parieto-occipital region.

**Figure 3:**
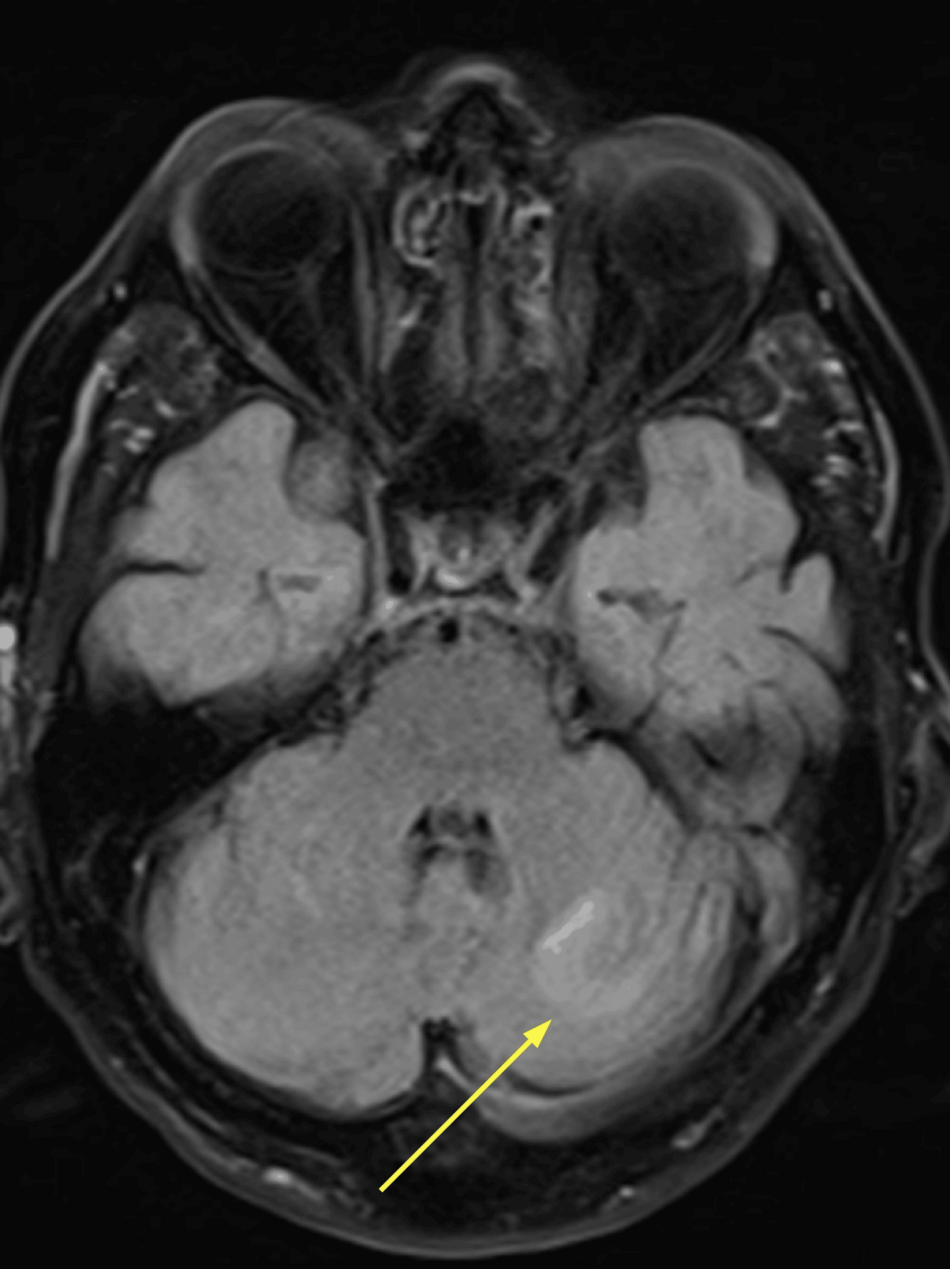
MRI brain: cerebellar section Within the superior right cerebellar hemisphere, a high signal appears to lie predominantly within the CSF spaces between the folia. Within the superior left cerebellar hemisphere is a 2 cm region of parenchymal FLAIR hyperintensity. The yellow arrow is pointing to the lesion in the left cerebellar hemisphere. CSF, cerebrospinal fluid; FLAIR, fluid-attenuated inversion recovery

An MRI brain performed two days later demonstrated focal enhancement and signal abnormality in the right parietal-occipital lobe with associated mass effect and bilateral cerebellar abnormalities. Differential diagnoses included malignancy (primary or metastatic) versus infarcts, though the subacute infarct pattern was considered less likely. Figure 2 shows the cerebral section in an MRI, and Figure 3 shows the cerebellar section.

The neuro-oncology multidisciplinary team (MDT) reviewed the imaging and noted well-defined abnormalities in both superior cerebellar hemispheres and the right occipital lobe. Differential diagnoses included subacute infarct or tumor. Follow-up MRI was advised in six weeks. The stroke MDT raised the possibility of leptomeningeal enhancement and an inflammatory or infectious process; primary glioma was considered less likely. A period of clinical stability followed initiation of dexamethasone at 8 mg twice daily, which was later tapered to 4 mg.

On day eight of admission, the patient developed fever, nausea, frequent vomiting, and subsequently aspirated, prompting initiation of intravenous antibiotics to cover aspiration pneumonia. Blood tests showed increasing inflammation markers. Table 2 shows blood results on day 8 of admission. 

**Table 2:** Blood results on day 8 of admission ALP, alkaline phosphatase; ALT, alanine transferase; CRP, C-reactive protein; GFR, glomerular filtration rate; WCC, white cell count

Parameter	Result	Normal range
Total WCC	23.6	4.0-10.0 * 10^9/L
Neutrophil count	22.3	2.0-7.0 * 10^9/L
Lymphocyte count	0.4	1.0-3.0 * 10^9/L
Hemoglobin	132	130-170 g/L
Platelet count	269	150-410 * 10^9/L
Serum urea	11.4	2.5-6.7 mmol/L
Serum creatinine	98	59-104 µmol/L
GFR (mL/min/1.73 m^2^)	57	-
Serum sodium	134	132-146 mmol/L
Serum potassium	5	3.5-5 mmol/L
Serum albumin	28	29-45 g/L
Serum ALT	21	0-35 U/L
Serum ALP	33	30-150 U/L
Serum bilirubin	15	0-17 µmol/L
CRP	160	0-9 mg/L

**Figure 4:**
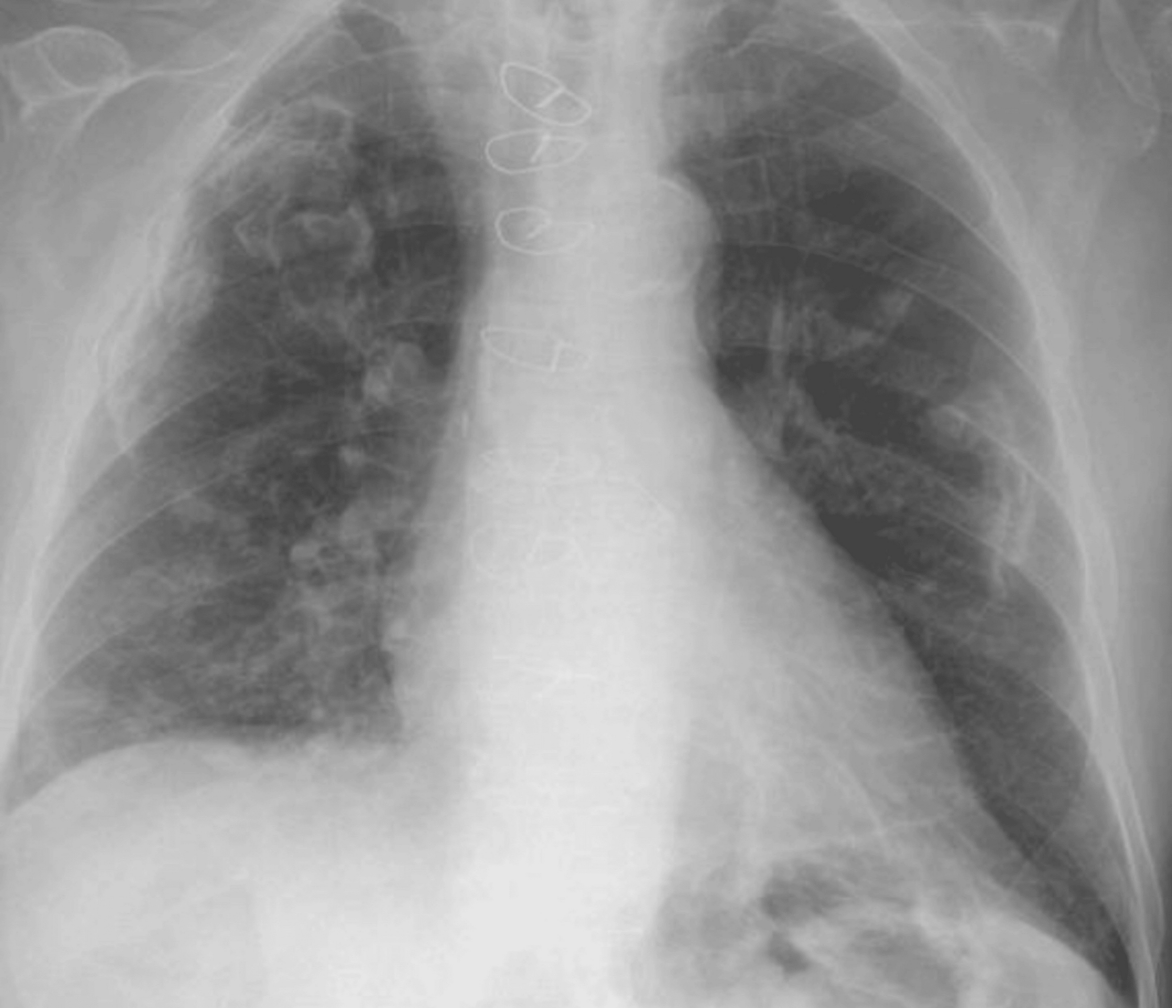
Chest X-ray Bilateral pleural plaques were noted. Sternotomy wires and aortic valve replacement were noted. There appears to be a new focus of consolidation in the right upper zone, medial to the large pleural plaque. The film is, however, significantly rotated, and this appearance may be due to a difference in the projection of the plaque. Old clavicle fractures were noted.

Chest X-ray revealed bilateral pleural plaques, an aortic valve replacement, and possible new consolidation in the right upper zone. Figure 4 shows the X-ray image.

Neurology consultation, on day 9 of admission, highlighted the progression of cerebellar signal changes on imaging, suggesting an infectious or inflammatory cerebellitis rather than malignancy; however, the patient remained afebrile with no antecedent viral illness or recent vaccinations. CRP remained low at admission, with no clear metabolic derangements to suggest a posterior reversible encephalopathy syndrome. Due to posterior fossa edema and effacement of the fourth ventricle, lumbar puncture was deferred for safety. An infectious and autoimmune panel, including HIV, syphilis, Lyme disease, viral polymerase chain reaction (herpes simplex virus, enterovirus, and varicella-zoster virus), erythrocyte sedimentation rate, antinuclear antibody, immunoglobulins, paraneoplastic antibodies, calcium, and lactate dehydrogenase, was sent and returned negative.

Following the neurology review, intravenous ceftriaxone and aciclovir were initiated in addition to ongoing treatment for aspiration pneumonia. This comprehensive antimicrobial strategy aimed to cover possible bacterial and viral CNS infections while addressing the pulmonary infection. He was continued on dexamethasone 4 mg IV twice daily, though escalation of steroids was avoided given the concurrent aspiration pneumonia.

**Figure 5:**
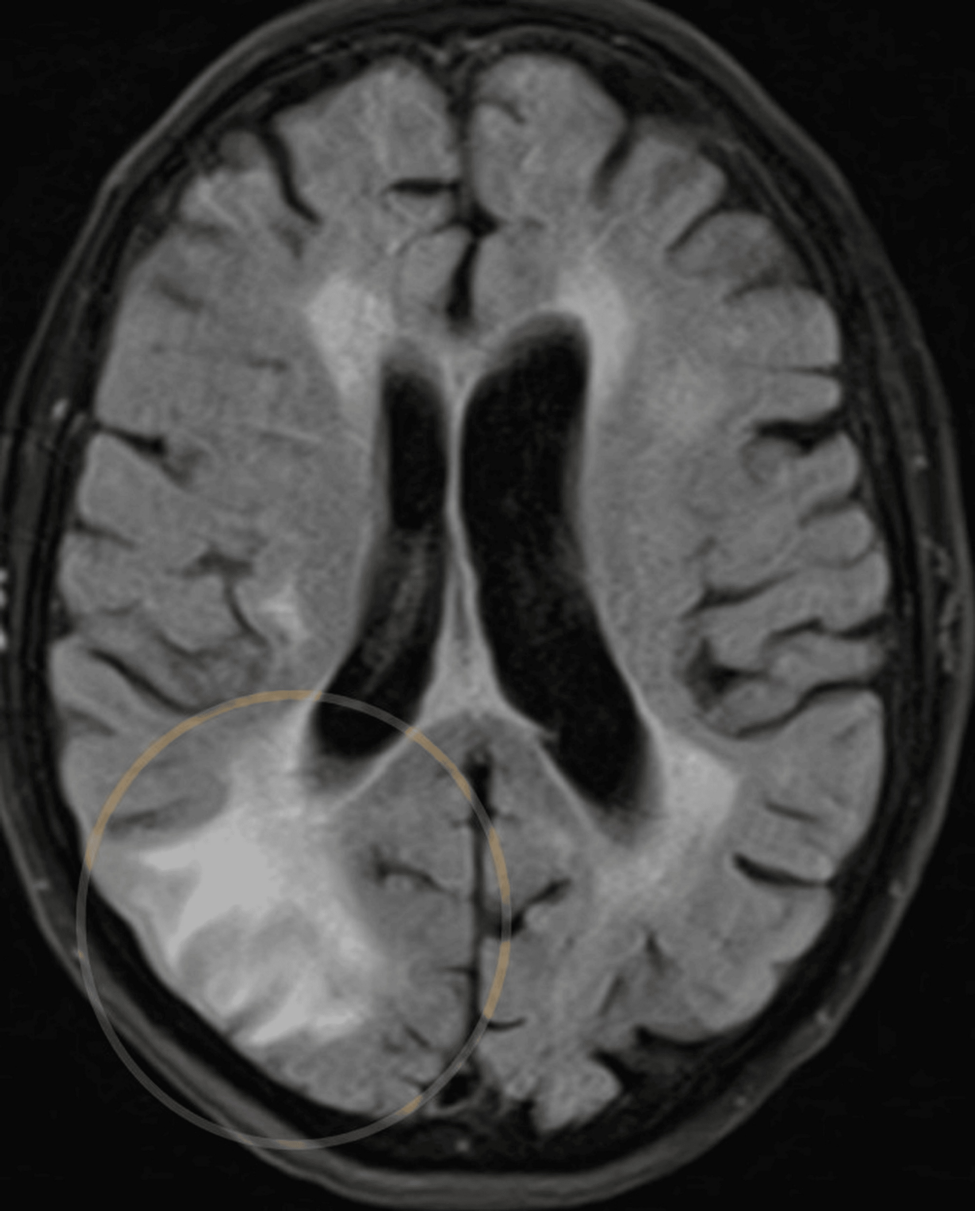
Repeat MRI brain on day 15 of admission: cerebral section MRI brain demonstrates ongoing abnormality in the right parieto-occipital region with evidence of evolution. Surrounding vasogenic edema is again noted, with mild increased extent of the edema medially compared to the previous MRI. The lesion is demarcated by a yellow circle.

**Figure 6:**
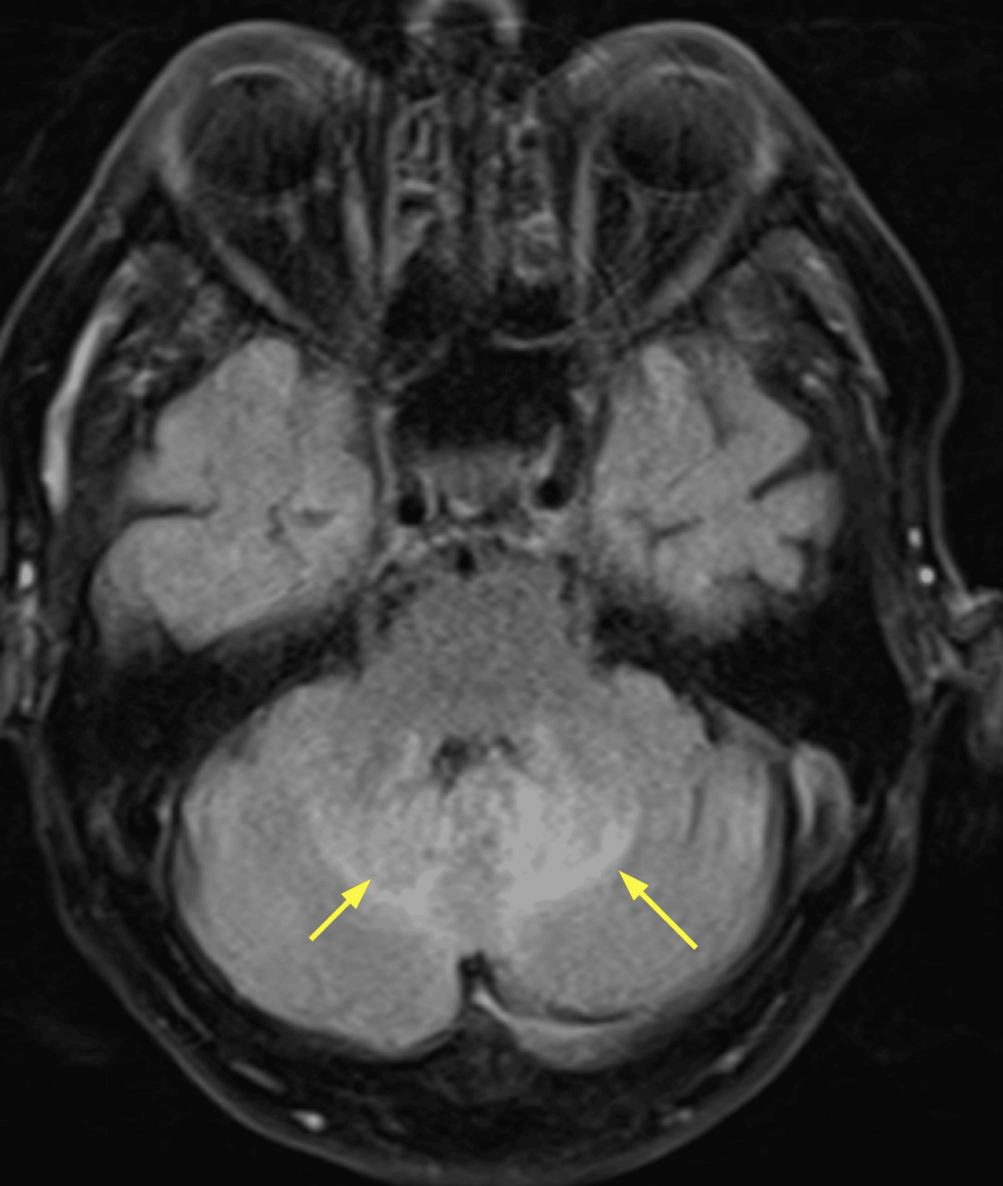
Repeat MRI brain on day 15 of admission: cerebellar section Ongoing changes within the cerebellum were noted, with high FLAIR signal persisting within the folia. There remains a distortion of the fourth ventricle. Two yellow arrows are pointing to these bilateral cerebellar lesions. FLAIR, fluid-attenuated inversion recovery

A repeat MRI on day 15 demonstrated further evolution of the right parieto-occipital lesion, increased vasogenic edema, and ongoing fluid-attenuated inversion recovery hyperintensity in the cerebellar folia. The rapid progression of imaging appearances made an infectious or inflammatory process more likely, with malignancy felt less probable. Figures 5, 6 show the repeated MRI brain images of cerebral and cerebellar sections.

**Figure 7:**
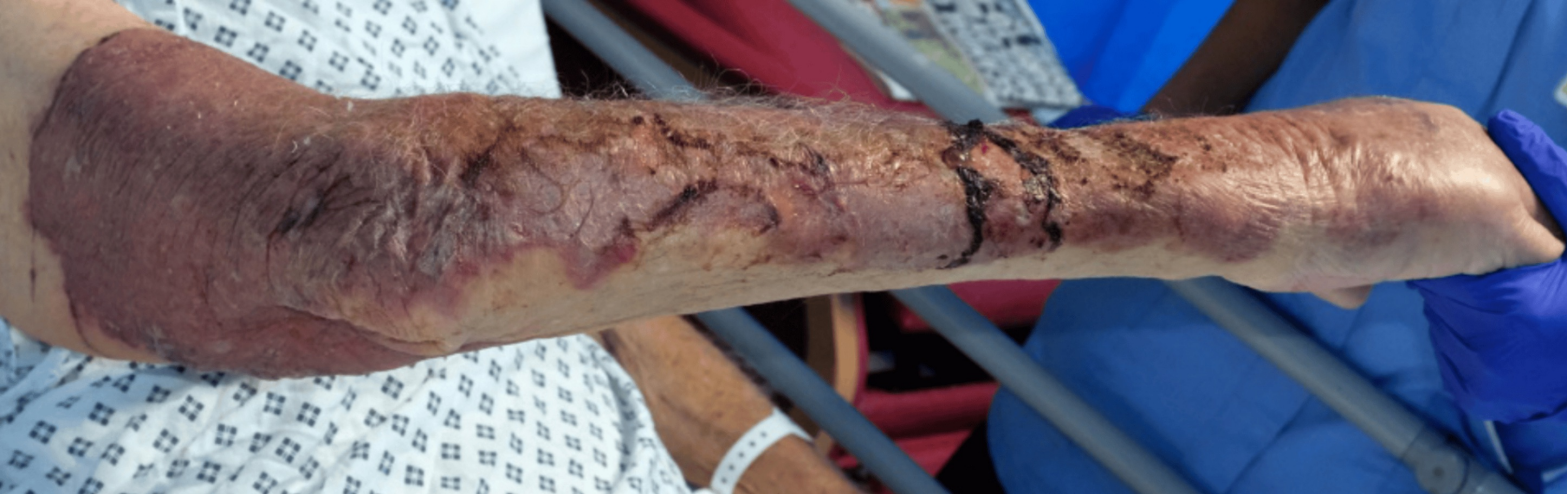
Upper limb cellulitis with areas of necrosis Cellulitis of the right upper limb was noted, extending from just above the right elbow to the right hand. The area is erythematous and well demarcated, mostly on the extensor surface of the upper limbs. Some areas of skin loss and black discoloration were noted, which may represent necrosis.

On day 23 of admission, the patient developed rapidly progressive skin changes on the right forearm, consistent with cellulitis. The area became painful, necrotic, and foul-smelling, raising suspicion for necrotizing cellulitis. Dermatological assessment confirmed extensive skin breakdown. Figure 7 shows the upper arm cellulitis.

Antibiotics were escalated to intravenous meropenem and clindamycin. A few days later, wound swabs showed heavy growth of *S. boydii*, confirmed on repeat swabs. Blood cultures remained negative. Susceptibility testing showed resistance to amphotericin and itraconazole but sensitivity to voriconazole.

Voriconazole and flucytosine were commenced following microbiology and national mycology reference laboratory input. A surgical opinion was sought for the debridement of the necrotic lesion but attempts to image the upper limb with MRI were unsuccessful due to patient intolerance. Retrospective review suggested that the initial gardening fall, involving muddy, bramble-laden ground, was the likely portal of cutaneous inoculation, with systemic corticosteroid use facilitating dissemination.

Despite appropriate antifungal therapy and supportive care, the patient’s condition continued to deteriorate, evolving into a disseminated fungal infection with multiorgan failure. Palliative measures were initiated on day 35 of admission, and all active interventions were withdrawn. He passed away on day 41.

A summary of the patient’s clinical course, investigations, and interventions is provided in Table 3.

**Table 3:** Timeline of key clinical events, investigations, and interventions

Timeline	Event
5 weeks before admission	Gardening fall leading to minor scratches
For 5 weeks	Confusion and gait disturbance
For 3 days before admission	Severe, predominantly posterior headache and slurred speech
Day 1	Admitted with headache, CT shows brain lesions → started dexamethasone
Day 3	1st MRI brain showing cerebellar abnormalities and cortical lesion
Day 8	Aspiration pneumonia → treated with IV antibiotics
Day 9	Neurology review: due to rapid progression, infection is more likely. Started ceftriaxone + acyclovir
Day 15	2nd MRI brain showing rapid progression of initial changes
Day 23	Necrotizing cellulitis on upper limb
Day 27	*Scedosporium boydii* isolated → started systemic antifungals
Day 35	End of life care. All active treatments were discontinued.
Day 41	The patient passed away

## Discussion

*S. boydii* is an opportunistic mold that causes a wide spectrum of infections, ranging from localized skin and soft tissue involvement to life-threatening disseminated disease [1]. In immunocompromised hosts, particularly those receiving corticosteroids or with underlying chronic conditions, the organism can invade deep tissues, including the CNS [1,2]. Such CNS involvement poses a significant clinical challenge due to its severity and diagnostic ambiguity. 

The present case demonstrates the diagnostic complexity of *S. boydii* CNS infections. Neuroimaging in these patients often reveals lesions that can be mistaken for neoplastic or vascular processes, as seen in our patient’s evolving cerebellar and parietal-occipital abnormalities. Such presentations have been well documented, with CNS manifestations most commonly following near-drowning incidents [3]. While traumatic inoculation is a recognized entry route, specific cases related to gardening injuries are less well documented.

Definitive diagnosis requires culture and, increasingly, molecular identification techniques, given the pathogen’s resistance profile [4]. In our case, the organism was isolated from a necrotizing skin infection, emphasizing the role of direct tissue sampling in establishing the diagnosis. This is consistent with prior reports highlighting that blood cultures are often negative, necessitating tissue biopsy for microbiological confirmation [1,4].

Voriconazole is the first-line therapy for *Scedosporium* infections and has shown efficacy even in CNS disease [1,5]. However, outcomes in disseminated infections remain poor, with mortality rates exceeding 70% in some series [2,6]. Surgical intervention may be beneficial in select cases with focal abscesses or necrotizing skin involvement, although this is not always feasible [5,7]. In our patient, despite initiation of voriconazole and flucytosine, clinical deterioration continued, reflecting the aggressive nature of disseminated *Scedosporium* infections.

This case underscores the importance of maintaining a high index of suspicion for rare fungal pathogens in immunocompromised patients with atypical neurological presentations and evolving CNS lesions. Early multidisciplinary collaboration, including infectious diseases, neurology, and microbiology expertise, is essential to guide diagnostic and therapeutic decision-making in these challenging cases.

## Conclusions

This case highlights the rare but serious potential for *S. boydii* to cause disseminated CNS infection in an immunocompromised patient following cutaneous inoculation. Despite treatment with voriconazole and flucytosine, the patient’s outcome was poor, reflecting the aggressive nature of invasive *Scedosporium* infections and the diagnostic challenges they pose. Clinicians should maintain a high index of suspicion for such fungal infections in immunosuppressed patients presenting with atypical neurological symptoms and evolving imaging findings, especially when there is a history of environmental exposure. Early tissue sampling, microbiological diagnosis, and multidisciplinary involvement are crucial to guide prompt and effective treatment.
